# Regulation of *Clostridium tetani* Neurotoxin Expression by Culture Conditions

**DOI:** 10.3390/toxins14010031

**Published:** 2022-01-02

**Authors:** Jeroen L. A. Pennings, Eric Abachin, Raphaël Esson, Hennie Hodemaekers, Antoine Francotte, Jean-Baptiste Claude, Céline Vanhee, Sylvie Uhlrich, Rob J. Vandebriel

**Affiliations:** 1 Centre for Health Protection, National Institute for Public Health and the Environment (RIVM), 3721 MA Bilthoven, The Netherlands; hennie.hodemaekers@rivm.nl (H.H.); rob.vandebriel@rivm.nl (R.J.V.); 2 Sanofi Pasteur, 1541 Avenue Marcel Mérieux, 69280 Marcy l’Etoile, France; Eric.Abachin@sanofi.com (E.A.); Raphael.Esson@sanofi.com (R.E.); Sylvie.Uhlrich@sanofi.com (S.U.); 3 Department of Chemical and Physical Health Risks, Sciensano, 14 Rue Juliette Wytsman, 1050 Brussels, Belgium; Antoine.Francotte@sciensano.be (A.F.); Celine.Vanhee@sciensano.be (C.V.); 4 IVIDATA Life Sciences, 79-83 Rue Baudin, 92300 Levallois Perret, France; Jean-Baptiste.Claude-ext@sanofi.com

**Keywords:** tetanus vaccine, next-generation sequencing, consistency approach, gene expression, targeted LC-MS/MS, 3Rs principles

## Abstract

Background: Ensuring consistency of tetanus neurotoxin (TeNT) production by *Clostridium tetani* could help to ensure consistent product quality in tetanus vaccine manufacturing, ultimately contributing to reduced animal testing. The aim of this study was to identify RNA signatures related to consistent TeNT production using standard and non-standard culture conditions. Methods: We applied RNA sequencing (RNA-Seq) to study *C. tetani* gene expression in small-scale batches under several culture conditions. Results: We identified 1381 time-dependent differentially expressed genes (DEGs) reflecting, among others, changes in growth rate and metabolism. Comparing non-standard versus standard culture conditions identified 82 condition-dependent DEGs, most of which were specific for one condition. The tetanus neurotoxin gene (*tetX*) was highly expressed but showed expression changes over time and between culture conditions. The *tetX* gene showed significant down-regulation at higher pH levels (pH 7.8), which was confirmed by the quantification data obtained with the recently validated targeted LC-MS/MS approach. Conclusions: Non-standard culture conditions lead to different gene expression responses. The *tetX* gene appears to be the best transcriptional biomarker for monitoring TeNT production as part of batch-to-batch consistency testing during tetanus vaccine manufacturing.

## 1. Introduction

The detoxified form of the *Clostridium tetani* neurotoxin (TeNT) is the essential component of (human) tetanus vaccines. The manufacturing process starts with the generation of stable and good manufacturing practice (GMP)-monitored toxin-producing *C. tetani* seed lots, which are then cultivated for days until the end of their growth phase. At this point, TeNT is harvested from the culture supernatant, purified, and inactivated by the addition of formaldehyde. The resulting toxoid is then mixed with an adjuvant, usually aluminum salts, prior to filling and packaging of the vaccine [1]. The final product is subsequently subjected to batch release, an extensive quality control testing performed by both the producer and an official control authority for batch release. In Europe, the latter role is carried out by Official Medicines Control Laboratories (OMCLs).

Many quality control assays for batch release have been used for decades and are historically part of Pharmacopeial Monographs from the European Pharmacopoeia (Ph. Eur.) or the United States Pharmacopeia (USP) along with World Health Organization (WHO) guidelines. These batch release methods often rely on animal models. Although animal-based potency and safety assays described within Ph. Eur. vaccine monographs have played a central role in safeguarding the quality of vaccines for decades, there are ongoing international efforts that look for alternative animal-free methods for vaccine quality control. To some extent, these have arisen from public and regulatory concerns about the ethical implications regarding animal testing [2]. The inherent variability of in vivo assays can also make them less suitable than appropriately designed in vitro assays for monitoring the consistency of production and for assessing the potential impact of manufacturing changes [3]. Finally, in modern vaccine development, production, and control, there is a better characterization of the product during development, improved optimization and standardization of the production process, and intensive in-process control and product monitoring with superior analytical tools and the use of quality systems such as GMP to guarantee consistency in both production and testing methods. Such an approach results in consistent vaccine batches with similar characteristics to those batches already shown to be safe and effective in the target population [4]. This has, along with other strategies, led to the development of the consistency approach. This approach relies on the strict application of a quality system and control of all steps along the production process, aimed at a production of final batches consistent with the criteria as defined in the marketing authorization, which should lead to a reduction in the number of animals used for the testing of biological products [4]. In the case of established inactivated bacterial vaccines such as tetanus, one could expect to see a large impact on animal testing [4,5].

One of the major critical points in the production of clostridial vaccines is the stable and reproducible production of high levels of toxins, as industrial TeNT production is sometimes hampered by low titers and occasional batch failures [6]. Quantitation of TeNT is traditionally carried out by an in vitro flocculation method that expresses the amount of toxin in terms of limit of flocculation (Lf) units [7,8,9,10]. The Lf method is based on binding the tetanus antigen and a specific antiserum, which produces a flocculent precipitate. However, the Lf test is known to suffer from considerable variation in output [11,12]. As an alternative in vitro approach to TeNT quantitation, various enzyme-linked immunosorbent assays (ELISAs) have been developed [12,13,14]. Although these ELISAs have proven to be highly specific and sensitive, they rely on the generation antibodies that require animal immunization [12,14,15,16,17] and they are generally only performed at the end of the production phase. Consequently, in accordance with the consistency approach and 3Rs principles, TeNT quantification for in-process product monitoring would benefit from fast animal- and antibody-free analytical tools that are readily applicable during the early production phases to track down a potential batch failure as early as possible.

The goal of this study is to identify RNA signatures related to consistent TeNT production during batch fermentation under manufacturing conditions, with a view to better monitoring the consistency of this manufacturing step.

## 2. Results

### 2.1. Growth

**Figure 1:**
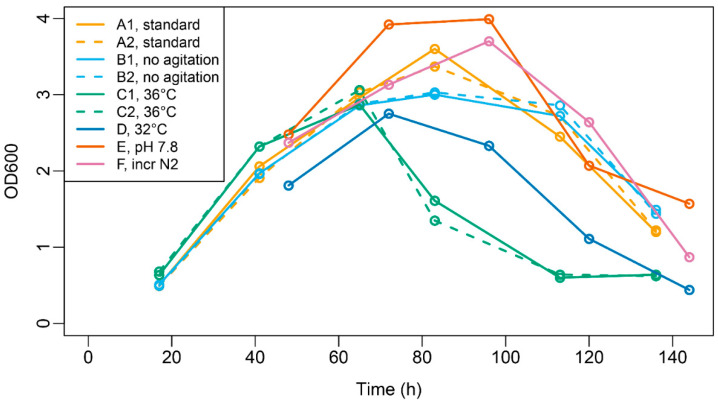
Optical density of batch cultures. Culture conditions are explained in Table 1.

*C. tetani* cultures were grown using simulated vaccine production processes under different small-scale batch fermentation conditions, comprising standard conditions and modifications thereof (Table 1). A comparison of the optical density (OD) curves showed that the OD values followed a similar course over time (Figure 1). The chosen sampling points (see Table 1 for details) comprised the early (exponential–linear), middle (stationary), and late (lytic) phases of growth and therefore spanned most of the batch culture.

### 2.2. Gene Expression Analysis

**Figure 2:**
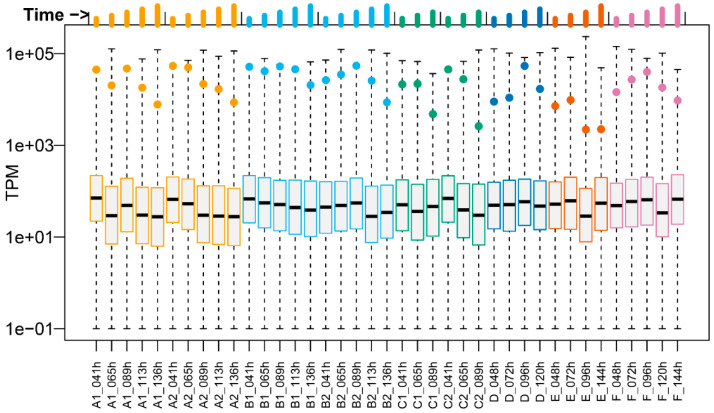
Expression of the tetanus neurotoxin gene (*tetX*) compared to the rest of the *C. tetani* genome. The expression of all genes per sample is shown as a box blot indicating transcripts per million (TPM) values. Boxes indicate the first quartile, median, and third quartile per sample, with the whiskers extending to minimum and maximum values. The *tetX* gene is indicated as a solid dot. Bar lengths at the top of the figure indicate time points. Culture conditions per sample are explained in Table 1.

Visualization of genome-wide transcriptional activity, as depicted in Figure 2, revealed that tetanus neurotoxin (*tetX*) gene expression was consistently high compared to the rest of the transcriptome, and this held for the majority of the bacterial growth conditions. In fact, in seven out of 39 samples the *tetX* gene had the highest expression level of any of the transcripts monitored. However, when the bacteria were grown either at 36 °C or pH 7.8, the *tetX* transcriptional activity was somewhat lower (Figure 2).

A comparison of genome-wide transcriptional profiles indicated that duplicate samples (i.e., grown under the same culture conditions and sampled at the same time point) were highly similar (median Spearman’s R = 0.967) and that differences in genome-wide transcriptional profiles generally were more pronounced between time points than across different culture conditions (median Spearman’s R = 0.916 for same condition at different time points, 0.924 for different conditions at same time point). Therefore, an ANOVA model that included time and culture condition as variables was used in our further analyses.

**Figure 3:**
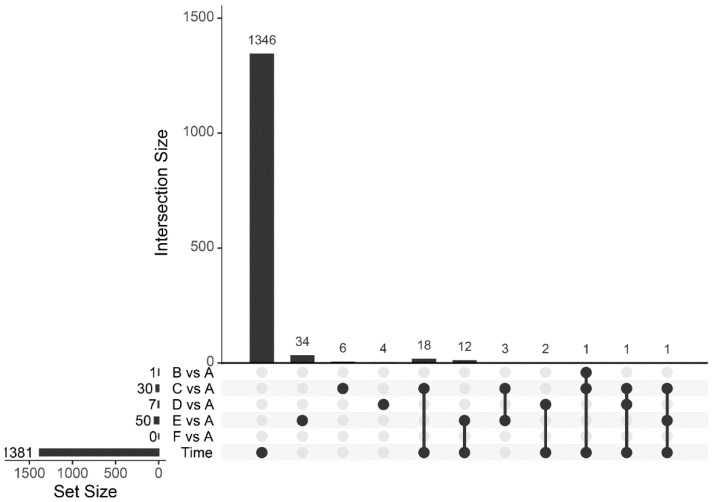
UpSet plot showing the overlap between differentially expressed genes by time and/or culture condition. Culture conditions are explained in Table 1.

Statistical analysis revealed that 1381 genes were differentially expressed over time (Figure 3). A list of these genes is available in Supplementary Table S1. Although the aim of our study did not include a detailed analysis of time-related gene expression changes, we found that the DEGs with the strongest down-regulation over time involved genes chiefly involved in nucleotide synthesis, transcription, and translation. DEGs with the strongest up-regulation over time were predominantly involved in metabolic processes and metabolite transport (Supplementary Table S1). Overall, these changes are consistent with slower growth and adaptations to nutrient depletion over time.

The number of DEGs by non-standard culture condition versus standard conditions was 1 for no agitation, 30 for 36 °C, 7 for 32 °C, 50 for pH 7.8, and 0 for increased nitrogen flow (Figure 3). There was relatively little overlap between these DEGs, so combining them resulted in a set of 82 DEGs, of which 35 were also differentially expressed over time (Figure 3). Overall, only six out of the 82 DEGs were differentially expressed under more than one culture condition. CDP-diacylglycerol-serine O-phosphatidyltransferase (CTC_RS06965) was down-regulated under conditions of no agitation and up-regulated at increased temperature (36 °C). Methyl-accepting chemotaxis protein (CTC_RS07835) was up-regulated at 36 °C and down-regulated at 32 °C. Additionally, three hypothetical proteins (CTC_RS05535, CTC_RS05630, CTC_RS05640) and a phage capsid protein (CTC_RS05540) were down-regulated at pH 7.8 and 36 °C.

**Figure 4:**
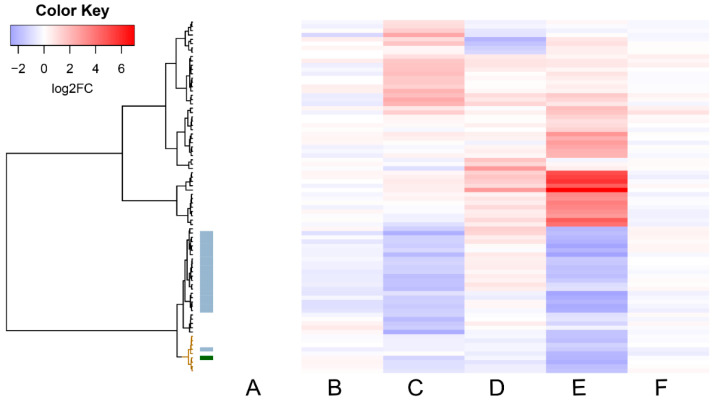
Heatmap showing the 82 genes identified as differentially expressed by at least one non-standard condition. Culture conditions are explained in Table 1. Expression values are group average fold changes compared to standard conditions, expressed on a log2-scale. The *tetX* gene is indicated by a green side bar, and prophage region genes by grey side bars. The gene cluster with the highest expression similarity to *tetX* is indicated in brown in the dendrogram.

A heatmap of the DEG expression changes showed the overall pattern of differences between the various culture conditions (Figure 4). From a vaccine manufacturing point of view, the most interesting DEG would be *tetX* (CTC_RS14060), as this gene codes for the neurotoxin used for vaccine manufacturing [18]. *tetX* was significantly (FDR < 5%) downregulated at pH 7.8 and also, albeit not significantly, at 36 °C (conditions E and C in Figure 4, respectively). A cluster of nine DEGs that were down-regulated at pH 7.8 showed expression patterns that were highly similar to those of *tetX* (Figure 4, brown cluster). Apart from *tetX*, these included acyl-CoA dehydrogenase (CTC_RS10685), amino acid permease (CTC_RS04695), ethanolamine utilization protein (CTC_RS11165), and five hypothetical proteins (CTC_RS05450, CTC_RS08030, CTC_RS08110, CTC_RS10695, CTC_RS13650). When adjacent clusters were included, we found a set of 20 DEGs for which the overall gene expression changes resembled those of *tetX* and that were located close together on the chromosome, rather than the plasmid on which the *tetX* gene is located. Additional BLAST alignment analysis indicated that these 20 genes are part of a prophage locus that corresponds to *Clostridium* phage phiCTC2A. This locus spans the *C. tetani* chromosome from positions 1,138,018 to 1,185,037 and contains 62 genes, including four down-regulated genes under both 36 °C and pH 7.8 (the aforementioned CTC_RS05535, CTC_RS05630, CTC_RS05640, CTC_RS05540). A full list of DEGs and their expression profiles is available in Supplementary Table S2.

### 2.3. Targeted LC-MS/MS Analysis

**Figure 5:**
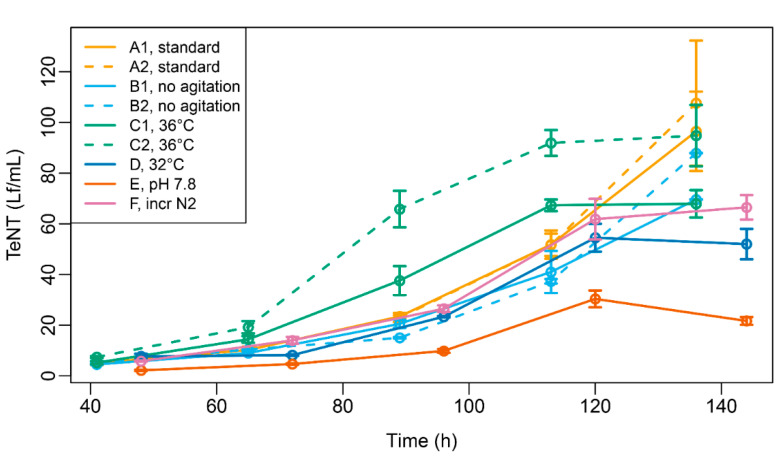
Targeted LC-MS/MS measurement of tetanus neurotoxin (TeNT) in culture medium during batch fermentation. Values are given as flocculation units per mL (Lf/mL). Error bars indicate standard deviations of triplicate measurements. Culture conditions are explained in Table 1.

To determine how gene expression changes in *tetX* would manifest on a protein level, we utilized the recently described and validated targeted LC-MS/MS methodology [19] to measure the amount of TeNT, the protein product of *tetX*, produced in the culture medium under those different circumstances. The results (Figure 5) show that the TeNT concentrations in culture medium increased over time. At the final time point, the highest concentrations were found for the standard conditions, which is in agreement with our expectations for a standardized and optimized production process. Although the TeNT concentration kinetics and final concentrations differed somewhat between culture conditions, the two most extreme conditions were those of 36 °C and pH 7.8. The amount of TeNT present in the medium increased more rapidly when the growth temperature was augmented and reached a plateau at lower concentrations (expressed in Lf/mL) compared to the harvesting phases under normal growth conditions (Figure 5, green and orange lines, respectively). A different phenomenon was observed at pH 7.8, where the TeNT concentrations were consistently lower across the entire time course than under any of the other conditions, with concentrations that were on average 43% of the standard conditions. This corroborates our gene expression findings of down-regulation of *tetX* at pH 7.8.

## 3. Discussion

The goal of this study was to identify RNA signatures related to consistent TeNT production using samples obtained from standard (conforming) and non-standard (non-conforming or stressed) culture conditions. To this end, gene expression analysis was performed on *C. tetani* cultures grown under different conditions. Each of these conditions differed from standard conditions by a change in one parameter that is typically carefully controlled during batch fermentation. Therefore, it may be expected that such non-standard conditions can be considered stressed conditions that would lead to gene expression changes.

As a method for gene expression analysis, RNA-Seq was used. The main strength of this method is the possibility to simultaneously quantify the number of mRNA transcripts for all genes—and even noncoding transcripts—in the genome. A relative disadvantage is the time needed for library preparation and sequencing, which would not be practical for future in-process monitoring settings. For identifying RNA signatures, the possibility to analyze all genes outweighs this disadvantage, whereas for in-process settings faster methods such as reverse transcription–polymerase chain reaction (RT-PCR) can be used on a small subset of genes.

Comparison of the gene expression data revealed that the majority (1381 out of 2706) of the *C. tetani* genes showed significant (FDR < 5%) expression changes over time, reflecting changes in growth rate and metabolism, among others. The overall aim of this work, however, was not to investigate changes during batch fermentation culture, but rather differences between culture conditions that could be informative parameters for monitoring vaccine manufacturing. Viewed in this way, the degree of regulation was much smaller. For some, but not all, culture conditions, significant DEGs were identified. Most of these DEGs were only significant (FDR < 5%) for one culture condition (Figure 3), and the heatmap (Figure 4) revealed qualitative as well as quantitative differences between the expression profiles for each of the various conditions. In other words, we did not identify a universal biomarker DEG for all stress conditions. However, from a vaccine manufacturing point of view, it is reassuring that *tetX* is one of the DEGs, as this gene codes for the tetanus neurotoxin [18]. Expression of *tetX* was significantly (FDR < 5%) down-regulated by the culture conditions pH 7.8 and (albeit not significantly) 36 °C, as well as by time. Despite its differential expression by culture conditions and time, expression of *tetX* was always high and relatively stable during and between cultures (Figure 2), which shows that growth phase and conditions only affected *tetX* expression to a limited extent. This contrasts with the findings described by Licona-Cassani et al. [6], which were later confirmed by Orellana et al. [20]. Licona-Cassani et al. found that *tetX* expression is triggered by the transition from amino acid consumption to peptide consumption, corresponding to a switch to a slower growth rate at around 10 h of culture. However, it should be noted that this switch would occur well before our first sampling point, the growth curve was not entirely similar to that in our study, their medium composition was slightly different than ours, and some other culture conditions may have been different, too. This makes it challenging to fully compare the findings of Licona-Cassani et al. with ours.

Several literature studies looked into the molecular regulation of *tetX* expression. The expression of *tetX* is mainly regulated by the BotR transcription factor (also known as TetR [21]), which is coded on the *C. tetani* plasmid immediately upstream from *tetX* [21]. More recently, it was discovered that there are several two-component systems or individual proteins that can influence *tetX* expression by binding to the *tetX* and/or *botR* promotor [22]. However, neither botR nor any of the factors mentioned in [22] showed significant differential expression between standard and non-standard cultures. This leaves the mechanism behind the observed down-regulation of *tetX* gene expression still open for further research.

The DEG heatmap (Figure 4) revealed that there was a cluster of DEGs with expression patterns that resembled those of *tetX* and that were significantly down-regulated by pH 7.8 and/or 36 °C. However, for most of these DEGs, little functional annotation is available, and the magnitude of their down-regulation was smaller than for *tetX* (Supplementary Table S2), which makes it difficult to assess their potential benefits as biomarkers in addition to, or instead of, *tetX*. A possible exception might be the DEGs that are encoded by the prophage locus. In other *Clostridium* species, as well as other bacteria, prophage genes can influence cellular lysis (which leads to the release of TeNT into the medium) as well as regulate toxin gene transcription [23,24]. In fact, Orellana et al. found that two genes (CTC_RS05470 and CTC_RS07675) located in different prophage insertion regions in the *C. tetani* genome clustered with the transcription of the *tetX* transcriptional regulator BotR [20]. It is therefore conceivable that further research on this prophage region may lead to new insights into TeNT regulation.

Taking into account all the findings described above, it is clear that growth phase and metabolic adaptations are major factors in *C. tetani* genome-wide expression regulation. At the culture time points (i.e., growth phases) sampled, *tetX* expression was consistently high, so it was either expressed from the beginning onwards in our medium, or *tetX* expression was triggered at a timepoint preceding our earliest sample point. Deviating (i.e., non-standard) culture conditions (especially higher pH and temperature) led to decreased *tetX* expression. As *tetX* has a high expression and sufficiently large dynamic range, it could be useful as an initial transcriptional biomarker for evaluating process consistency and TeNT yields in large-scale batch fermentation cultures. Other DEGs identified in this study might also be useful for assessing the extent of standard and/or non-standard culture conditions or, alternatively, gaining insight into the cellular adaptations (co)occurring during such non-standard culture conditions. Finally, since our findings for *tetX* under the described conditions were also consistent with the findings at the TeNT protein level, future manufacturing batch monitoring could be based on RNA as well as protein analysis. A choice between RNA- or protein-based batch consistency monitoring will likely depend on technical factors as well as regulatory acceptance. In this respect, it may be noted that both RNA-Seq and LC-MS/MS require specialized equipment and highly trained personnel and therefore other methodologies for determining RNA and/or protein abundance, such as RT-PCR, may be preferential for implementation in vaccine manufacturing processes.

## 4. Materials and Methods

### 4.1. Samples

*C. tetani* (strain A) batch cultures were grown at Sanofi-Pasteur using simulated vaccine production processes under different small-scale batch fermentation conditions in a 250 mL culture using Ambr 250 High Throughput Vessels (Sartorius, Göttingen, Germany), using the medium type as described in [25]. The cultures were performed during two studies; the second study served as a confirmation as well as an expansion of the first study. A total of nine cultures were used for gene expression analysis, representing standard conditions (34 °C, pH 7.3, 150 rpm stirring, 30 mL/min N_2_ flux in headspace) and five non-standard (stress) conditions that differed from standard conditions by changing one of these parameters, namely, no agitation, higher (36 °C) and lower (32 °C) temperature, higher pH (pH 7.8), and an increased N_2_ flux in the headspace (Table 1). In the first study, cultures were performed in duplicate. Bacterial samples were taken at five time points when possible.

A schematic overview of the sampling conditions is given in Table 1. In addition to the nine cultures described in Table 1, additional cultures were performed that either did not show any growth (pH 6.0, study 1) or that, based on, e.g., growth profile and cellular morphology, were considered too similar to normal conditions (pH 6.8 and increased agitation, study 2). These cultures were not used for gene expression analysis.

### 4.2. RNA Isolation and Sequencing

RNA extraction and purification were performed at Sanofi Pasteur. For RNA isolation, 10 mL samples of cell suspension were collected and centrifuged (5 min at 4500× *g*). After supernatant removal, the cell pellet was put in suspension with 500 μL of fresh medium. For RNA preservation, 1 mL of RNAprotect Bacteria Reagent (Qiagen, Hilden, Germany) was added. After homogenization, samples were stored at 5 °C for 5 min to 1 h and then stored at −70 °C until RNA extraction. After thawing, 10^9^ cells were collected for RNA extraction. The cell content was not enough for certain samples, and in this case the totality of the sample was used. RNA extraction and purification were performed by using the RiboPure™-Bacteria Kit from Life Technologies (Carlsbad, CA, USA) (ref. AM1925) and the RiboPure Bacteria procedure (Part Number 1925M Rev. D). Briefly, the protocol includes the following steps: The RiboPure-Bacteria method disrupts bacterial cell walls by beating cells mixed with RNAWIZ and 0.1 mm zirconia beads in a vortex adapter for 10 min. The lysate is then mixed with chloroform and centrifuged to form three distinct phases; the upper aqueous phase contains RNA. The RNA is then diluted with ethanol and bound to a silica filter. The RNA bound to the filter is washed to remove contaminants, and eluted in a low-ionic-strength solution. Post-elution removal of contaminating genomic DNA was done with Ambion (Carlsbad, CA, USA) DNA-free™ Reagents, and removal of the DNase I and divalent cations in the buffer was done using DNase Inactivation Reagent. As with all glass fiber filter purification methods, 5S ribosomal RNAs and tRNAs are not quantitatively recovered using the RiboPure-Bacteria Kit. Prior to storage, RNA QC was performed to determine the concentration and the integrity (RNA integrity number (RIN)). Purified RNA samples were stored at −70 °C and later shipped to RIVM. Total yield per sample varied from 2 to 30 µg of RNA for samples that were used in subsequent steps, with an average of 14 µg. Samples with an RNA yield of 1 µg or less were not used because this amount was insufficient for further analysis.

For the first study, RNA-Seq was performed at RIVM. A total of 26 samples were used for RNA-Seq; these were processed in two sequencing runs in such a way that samples from duplicate cultures were sequenced in the duplicate runs. Library preparation was performed using the TruSeq Stranded mRNA Library Prep kit (Illumina, cat.nr. 20020594, San Diego, CA, USA). Additional reagents used were the Ribo-Zero rRNA Removal Kit for Gram-Positive Bacteria (Illumina, cat.nr. MRZGP126), RNA Clean & Concentrator-5 (BaseClear, cat.nr. Zymo R1015, Leiden, the Netherlands), and the Baseline-ZERO DNase (Immunosource, cat.nr. Epicentre DB0715K, Schilde, Belgium). The library preparation PCR step used 15 cycles. Libraries were pooled and 1.3 mL (1.9 pM) combined with 1% PhiX Control v3 (Illumina, cat.nr FC-110-3001) were sequenced using Illumina NextSeq equipment and the NextSeq 500/550 High Output Kit v2.5 (cat.nr. 20024906) and 75 cycles.

For the second study, RNA-Seq was performed at BaseClear, using their Illumina-based protocols for bacterial RNA-Seq. A total number of 13 samples was used for RNA-Seq. FASTQ raw data were sent to RIVM via file transfer protocol (FTP) for further analysis.

### 4.3. Data Analysis

Technical quality control of the RNA sequencing data was performed in a Linux environment using a pipeline developed in-house containing open-source tools such as FastQC (version 0.11.5, Babraham Institute, Cambridge, UK) and MultiQC (version 1.5, Stockholm University, Stockholm, Sweden). This quality control step comprised aspects such as basecalling accuracy, GC content, N content, sequence length distribution, and adapter content.

Next, NGS reads were trimmed to the first 50 nucleotides and mapped to the genome of *C. tetani* using Bowtie2 software (version 2.2.4, University of Maryland, College Park, MD, USA) at standard settings. Mapping was done using the published reference genome sequence of the E88 strain [26], which consists of a chromosome (accession number NC_004557.1) containing 2620 coding genes and a plasmid (accession number NC_004565.1) containing 86 coding genes, including *tetX*. All genome data used were downloaded from NCBI. The E88 genome is practically identical to that of the A strain [27]. Bowtie2 alignments were processed by Samtools (version 1.1, Sanger Institute, Cambridge, United Kingdom) to make a count table, which was further analyzed using Microsoft Excel (Microsoft, Redmond, WA, USA) and R (version 3.5.1, R Foundation for Statistical Computing, Vienna, Austria) statistical software.

Normalization and statistical analysis were done in R statistical software using additional R packages “gplots,” “seqinr,” “UpSetR,” and “viridis” from CRAN (https://cran.r-project.org/ accessed on 28 November 2021) and “DESeq2” and “limma” from Bioconductor (https://bioconductor.org/ accessed on 28 November 2021). For the visualization of genome-wide gene expression, data were transformed to transcript per million. For all other visualizations and statistical analysis, a variance stabilizing transformation (vst) was applied. Gene expression values were compared using ANOVA [28] and a generalized linear model (GLM) [29] that included time, culture condition, and study number as parameters. P-values were adjusted for multiple testing by calculating the false discovery rate (FDR) [30]. FDR values < 5% were considered significant to decide which genes were differentially expressed. Data were visualized as a heatmap using hierarchical clustering with Euclidean distance and Ward linkage.

### 4.4. Targeted LC-MS/MS Analysis

The initial processing steps of cell suspension aliquots destined for targeted LC-MS/MS protein analysis were performed at Sanofi Pasteur. Samples for these analyses were taken in parallel to culture samples for gene expression analysis. Five mL cell suspensions samples were centrifuged (5 min at 4000× *g*) and the supernatants filtered through a 0.22 µm filter to ensure no residual bacteria were present. Next, 110 µL of 10 X protease inhibitor mixture (Roche, Basel, Switzerland) were added to 1 mL of each supernatant, followed by vortexing, storage at ≤−70 °C, shipment to Sciensano, and subsequent storage at temperatures ≤−70 °C until further processing.

A stock solution of 25 Lf/mL TeNT, used as an external calibrant as described by Francotte et al. [19], was generated with the extracellular medium from non-TeNT-producing bacteria (to represent the sample matrix) and purified TeNT. Stable heavy isotope-labeled versions of the two internal standard peptides (IS), belonging either to the light chain (Lc) or the heavy chain (Hc) of TeNT, were generated by Thermo Fisher Scientific (Waltham, MA, USA). ULC-MS grade acetonitrile and methanol were purchased from Biosolve (Valkenswaard, The Netherlands). Water was obtained using a Milli-Q Gradient A10 system (Millipore, Billerica, MA, USA). Analytical-grade formic acid was purchased from Merck (Darmstadt, Germany), whereas ammonium bicarbonate, dithiothreitol, iodoacetamide, and Tween-20 were obtained from Sigma-Aldrich (St. Louis, MO, USA). Sequencing-grade modified trypsin from Promega (Madison, WI, USA) was used as the digestion enzyme. All reactions were performed in Eppendorf LoBind protein tubes (Eppendorf, Hamburg, Germany).

The amount of TeNT present in the samples was assessed by means of a targeted LC-MS/MS method that was recently validated for the quantification of TeNT present in bacterial media during vaccine production [19]. Briefly, 50 µL of each aliquot were transferred to a 1.5 mL Eppendorf LoBind tube and 450 µL of cold methanol (stored at −20 °C) were added, followed by vortexing and incubation for 20 min at −20 °C. Next, the tubes were centrifuged at 20,000× *g* for 5 min and 450 µL of supernatant were removed. The pellet was washed once with methanol prior to the drying of the samples at 60 °C and subsequent incubation at −20 °C for at least two hours. The sample was then reconstituted with 50 µL of PBS +0.1% (*v*/*v*) Tween-20 and underwent the addition of the control peptides (IS), reduction with dithiothreitol, and alkylation of the cysteines by iodoacetamide prior to the addition to a final concentration of 0.1 M ammonium bicarbonate pH 8.0 and 5 µg of trypsin per sample. The digest was incubated overnight at 37 °C and the reaction was stopped by adding 10 µL of a 10% (*v*/*v*) formic acid solution, followed by a vacuum concentration step (5 h at 45 °C). The pellet was first incubated at −20 °C for at least two hours prior to reconstitution with 500 µL of 1% (*v*/*v*) formic acid solution. After digestion, the peptides were centrifuged at 20,000× *g* for 5 min and the supernatant was transferred to injection vials. The measurements were performed on a UHPLC device for chromatographic separation followed by detection using tandem mass spectrometry (MS/MS) in multi-reaction mode. For each TeNT quantification experiment, a calibration curve was generated using the stock solution of TeNT for conversion to Lf/mL units. The detailed LC-MS/MS conditions and validation data were described in [19]. All samples were prepared and analyzed in triplicate and standard deviation values were calculated from the TeNT concentration values.

TeNT concentrations for standard, no agitation, and 36 °C conditions were previously reported as part of the methodology description and validation [19]; TeNT data for the other conditions (32 °C, pH 7.8, increased N_2_ flux) have not been reported before.

## Figures and Tables

**Table 1:** Batch culture conditions and sampling.

Culture	Study	Culture Conditions	Sample Time Points (h) ^1^
A1, A2	1	Standard ^2^	41, 65, 89, 113, 136
B1, B2	1	As standard but no agitation	41, 65, 89, 113, 136
C1, C2	1	As standard but 36 °C	41, 65, 89
D	2	As standard but 32 °C	48, 72, 96, 120
E	2	As standard but pH 7.8	48, 72, 96, 144
F	2	As standard but 60 mL/min N_2_ flux	48, 72, 96, 120, 144

^1^ For missing sample time points, samples were taken but insufficient RNA was obtained for RNA-Seq analysis. ^2^ Standard conditions comprise 34 °C, pH 7.3, 150 rpm stirring, 30 mL/min N_2_ flux in headspace.

## Data Availability

The raw data and metadata from this publication are available at GEO (www.ncbi.nlm.nih.gov/geo/ accessed on 28 November 2021) under accession number GSE182408.

## Supplementary Materials

The following are available online at https://www.mdpi.com/article/10.3390/toxins14010031/s1, Table S1: differentially expressed genes by time, Table S2: differentially expressed genes by culture condition.
